# Object Tracking Algorithm Based on Dual Color Feature Fusion with Dimension Reduction

**DOI:** 10.3390/s19010073

**Published:** 2018-12-25

**Authors:** Shuo Hu, Yanan Ge, Jianglong Han, Xuguang Zhang

**Affiliations:** 1 School of Electrical Engineering, Yanshan University, Qinhuangdao 066004, China; hus@ysu.edu.cn (S.H.); sherrygyn@stumail.ysu.edu.cn (Y.G.); hanjianglong@stumail.ysu.edu.cn (J.H.); 2 School of Communication Engineering, Hangzhou Dianzi University, Hangzhou 310018, China

**Keywords:** feature fusion, self-adaptive feature fusion, principal component analysis, visual tracking, correlation filter

## Abstract

Aiming at the problem of poor robustness and the low effectiveness of target tracking in complex scenes by using single color features, an object-tracking algorithm based on dual color feature fusion via dimension reduction is proposed, according to the Correlation Filter (CF)-based tracking framework. First, Color Name (CN) feature and Color Histogram (CH) feature extraction are respectively performed on the input image, and then the template and the candidate region are correlated by the CF-based methods, and the CH response and CN response of the target region are obtained, respectively. A self-adaptive feature fusion strategy is proposed to linearly fuse the CH response and the CN response to obtain a dual color feature response with global color distribution information and main color information. Finally, the position of the target is estimated, based on the fused response map, with the maximum of the fused response map corresponding to the estimated target position. The proposed method is based on fusion in the framework of the Staple algorithm, and dimension reduction by Principal Component Analysis (PCA) on the scale; the complexity of the algorithm is reduced, and the tracking performance is further improved. Experimental results on quantitative and qualitative evaluations on challenging benchmark sequences show that the proposed algorithm has better tracking accuracy and robustness than other state-of-the-art tracking algorithms in complex scenarios.

## 1. Introduction

Visual object tracking is a very important branch of computer vision, and has been widely used in many fields, such as video intelligent traffic monitoring, robotics, surveillance, and human–computer interactions [1,2,3,4,5]. In recent years, discriminant tracking methods have gradually come to occupy a dominant position by using both target information and the background information around the target, in which tracking-by-detection methods [6,7,8,9] have provided excellent tracking performance. Among the existing tracking-by-detection methods, Correlation Filter (CF)-based tracking methods have attracted great attention, and have been widely used in visual target tracking, due to the computational cost characteristics of correlation operations through fast Fourier transformation (FFT) reduction algorithms in the frequency domain. In the MOSSE algorithm proposed by Bolme et al. [6], the first introduces a CF into the field of visual object tracking, which achieves real-time tracking with a speed of 669 frames per second. Furthermore, Henriques et al. proposed the CSK [7] tracker, which made a breakthrough for CF-based tracking algorithm in the field of tracking; by cyclic shifting, the sparse sampling is turned into dense sampling and combined with the Fourier transform, which greatly reduces the computational complexity. Moreover, in 2014, Henriques et al. proposed a KCF [8] tracker. They added a histogram of an oriented gradient (HOG) feature to CSK, instead of the previous Gray feature. In the same year, Danelljan et al. proposed the DSST [9] tracker, based on the KCF algorithm; a scale-dependent filter was added to estimate the target scale. However, the above methods only extract the gray features of the image, or convert the gray features into HOG features without considering the color information in the video, thus limiting the tracking accuracy of the algorithm, where in complex tracking scenarios, the tracking robustness of the target is poor.

Color measurements are robust for illumination, shadow, shading, specularities, and object geometrical variations, and have successfully been applied to image classification [10,11] and action recognition [12]. It has been proved that sophisticated color features can provide excellent performance for object recognition and detection. At the same time, the color feature is insensitive to the change of image size, orientation, rotation, and scale. Thus, it has a certain stability. In the aspect of color feature extraction, Swain and Ballad first proposed the color histogram [13]. Because of its simple and effective characteristics, this has become the most commonly used method for extracting color features. The Mean Shift algorithm [14] is a non-parametric kernel density estimation algorithm based on color kernel density estimation. Using the color histogram as an input feature, the candidate region with the largest similarity to the target probability density function is solved to achieve target tracking. In 2014, Danelljan et al. proposed the Color Name (CN) [15] tracker. In the field of computer vision, the operations of CN associate RGB with language color labels, mapping RGB values to 11-dimensional color representations. Using Principal Component Analysis (PCA) dimensionality reduction technology can reduce the 11 dimensions to two dimensions, which reduces the complexity of the algorithm, improves the computing speed, and promotes the wide application of color features in the target-tracking field. In 2015, the DAT [16] tracker used color trackers to distinguish between targets and backgrounds to achieve real-time tracking of online targets. Because a single color feature cannot give consideration to both local and global information in a complex tracking scene, it is susceptible to external interference, which leads to target-tracking drift or failure. The fusion of multiple color features can improve the tracking accuracy and robustness. The convenience of color feature reduces the computational costs of feature extraction. Many feature fusion algorithms use color features, e.g., [17,18,19,20]. In 2016, Bertinetto et al. proposed the Staple [21] tracker, which combines color features. This tracker greatly alleviates the influence of deformation on the tracking task, makes up for the lack of a single feature, and improves the robustness of the algorithm to deformation. In 2017, ECO [22] achieved excellent tracking performance by incorporating the CNN feature, HOG feature, and CN color feature. Additionally, in 2018, CVPR’s excellent algorithm, STRCF [23], incorporated spatial and temporal regularization into the DCF framework, and fused HOG features and CN color features in the CF framework. This algorithm is more robust to occlusion and can be very good for large changes in appearance.

In this work, we propose a Correlation Filter based tracker using a dual color feature fusion strategy, which improve tracking performance. This is motivated by the observation that the fusion color features alleviates the influence of deformation and occlusion. More specifically, we extract the CN features to overcome illumination variance, and we use the CH features to reduce the loss of tracking accuracy that occurs as a consequence of deformation and occlusion. In the tracking phase, a huge challenge is how to determine the proportion of each feature response. A parameter tuning task with a large workload is used for the traditional tracking algorithm, and through the constant tuning of parameters to find the algorithm it can make characteristic responses that are coefficient with the strongest generalization ability. To solve the above problems, we propose a method to adaptively adjust the characteristic response coefficient, considering the target scene, which reduces the tuning process in the fusion feature response stage. In order to further improve the performance of the algorithm, this paper performs PCA dimensionality reduction on the scale, based on the fusion of two-color features. The dimensionality reduction strategy is inspired by the fDSST [24] tracker. The computational cost of the DSST is dominated by the FFT. The training and detection steps require one FFT per feature dimension. In order to reduce the required number of FFT computations, the scale dimension is reduced to 17 dimensions by using a PCA dimension-reduction strategy. This strategy can reduce the high dimension to a low dimension, reduce the redundant calculation, and improve the operation speed and accuracy. We validate the proposed tracking method through quantitative and qualitative evaluations on challenging benchmark sequences. The benchmark sequences are sampled from both the fixed camera and moving platform. The experimental results show our method outperforms other CF-based trackers using non deep features and perform a real-time tracking.

The rest of this paper is arranged as follows: in Section 2, we describe the framework of the CF-based tracking algorithm. In Section 3, we describe the proposed tracking algorithm. Section 3.1 and Section 3.2 describe the CN features and the color histogram features, respectively. Section 3.3 introduces the color fusion strategy, while Section 3.4 introduces the scale reduction strategy. Section 4 provides experimental evaluation and analysis. Section 5 summarizes the paper and points out future research directions.

## 2. Correlation Filter (CF) Tracking

The CF-based tracking algorithm [6,7,8,9] is a multiple instance learning process. The basic samples are cycle-shifted by a permutation matrix, and the target region is densely sampled to obtain a large number of samples to train the classifier. By using a regularized least-squares classifier for a single image block-learning target, a kernel function is used to calculate the candidates. Using the similarity between the candidate region and the target region, the region with the maximum response is selected as the new tracking target, and the Discrete Fourier Transform is used to improve the running speed of the algorithm. The CF-based tracking algorithm mainly consists of three parts: classifier training, object detection, and parameter update.

### 2.1. Classifier Training

The CF is trained by ridge regression. The ridge regression problem can be transformed into a regularized least squares problem. For all training samples, *X*, and the expected output, *Y*, the classifier weight ω is solved by the optimization objective function (1). The purpose of training is to obtain a filter ω to represent the target model:

(1) $$\omega =\arg\underset{\omega }{\min}(\sum_{i,j}|<\phi (x_{i,j}),\omega >-y(i,j){|}^{2}+\lambda <\omega ,\omega >)$$

where, $x_{i,j}$ is the training sample obtained by a circulant shift; $y_{i,j}$ is the $x_{i,j}$ Gaussian label; ω is the weight coefficient; λ is a regularization parameter; and ϕ is the mapping to the high-dimensional feature space induced by the kernel K. Therefore, the solution of (1) can be expressed as:

(2) $$\omega =\sum_{i,j}\alpha (i,j)\phi (x_{i,j})$$

where *ω* is a linear low-dimensional spatial weight coefficient and α is mapped from a kernel function to a nonlinear high-dimensional space coefficient, which is obtained by Equations (1) and (2):

(3) $$A=F(\alpha )=\frac{F(y)}{F(k(x,x))+\lambda }$$

where *F*(.) is the Discrete Fourier Transform (DFT) operator and k(x,x) is the kernel function, where the idea of the kernel function is to map a linear indivisible problem in low dimensionality to a high-dimensional space through a kernel function, making the problem linearly separable in high-dimensional space. Suppose that *H* is a certain kind of feature space; if there is a certain mapping ϕ(x):x→H, the kernel function satisfies the inner product $\phi^{T}(x)\phi (x^{\prime })=k(x,x^{\prime })$.

### 2.2. Object Detection

The detection sample is the image block *z* of the same position in the next frame, and the classifier responds to the output:

(4) $$\hat{y}=F^{-1}(A⊗F(k(z,\hat{x}))$$

where ⊗ denotes the convolution operation; $F^{-1}$ denotes an Inverse Fourier transform; and $\hat{x}$ denotes a target appearance model for online learning.

The position of the maximum response of all test sample response vectors $\hat{y}$ to the predicted position of the object was found.

### 2.3. Parameter Update

To achieve object tracking that is robust to appearance changes, it is very important that the target model is updated over time. The CF-based tracking algorithm generally uses linear interpolation to update the target-apparent model $\hat{x}$ and the classifier coefficient A. The formula is updated as follows:

(5a) $${\hat{x}}_{t}=(1-\gamma ){\hat{x}}_{t-1}+\gamma {\hat{x}}_{t}$$

(5b) $${\hat{A}}_{t}=(1-\gamma ){\hat{A}}_{t-1}+\gamma {\hat{A}}_{t}$$

where γ denotes the learning rate; $\hat{x_{t}}$ denotes the *t*-th frame target-apparent model; and ${\hat{A}}_{t}$ denotes the *t*-th frame classifier coefficient.

## 3. Proposed Algorithm

**Figure 1:**
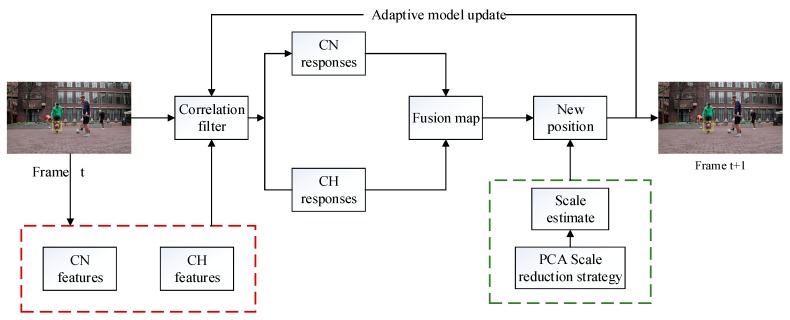
Framework of the proposed tracking method.

The framework of the proposed tracking method is shown in Figure 1. The framework of the algorithm can be roughly divided into five parts: the extraction of CN features and CH features; the dual color fusion of the CN response and the CH response that is obtained by the CF-based tracking algorithm; the use of the fusion map to estimate the position of the target object; scale estimation with the PCA dimension reduction technology; and the adaptive model update strategy.

### 3.1. Color Name (CN) Feature

The CN feature was proposed by Danelljan et al. [15]. The essence of the CN algorithm is to extend CSK by color attributes. An adaptive dimension reduction method is proposed to reduce 11-dimensional color features to two dimensions and to reduce the complexity of high-dimensional calculations.

RGB is mapped to 11 basic color attributes of black, blue, brown, gray, green, orange, pink, purple, red, white, and yellow. The original 11-dimensional color is reduced to a 2-dimensional main color attribute by using an adaptive dimensionality reduction technique. A suitable reduced-dimension map for the current frame *t* is found by minimizing Equation (6):

(6) $$\eta_{tot}^{t}=\alpha_{t}\eta_{data}^{t}+\sum_{j=1}^{t-1}\alpha_{j}\eta_{smooth}^{j}$$

where $\eta_{\mathrm{data}}^{t}$ is the data item that only depends on the current frame and $\eta_{smooth}^{j}$ is the smooth term related to the number of frames of weight *α*_1_, …, *α_t_*.

The appearance ${\hat{x}}^{t}$ of $D_{1}$ dimensional learning finds a projection matrix, $B_{t}$, of $D_{1}\times D_{2}$ on a standard orthogonal basis, and a new $D_{2}$ dimension appearance feature ${\tilde{x}}^{t}$ is calculated by linear mapping ${\tilde{x}}^{t}\left(i,j\right)=B_{t}^{T}{\hat{x}}^{t}\left(i,j\right),\forall i,j$. The data item consists of the reconstruction error of the current appearance as follows:

(7) $$\eta_{\mathrm{data}}^{t}=\frac{1}{MN}\sum_{i,j}{\left\|{\hat{x}}^{t}(i,j)-B_{t}B_{t}^{T}{\hat{x}}^{t}(i,j)\right\|}^{2}$$

The minimization of the data item (7) is a process of PCA dimensionality reduction on the current appearance. To obtain a more robust projection matrix, a smoothing term is added to Equation (6), as follows:

(8) $$\varepsilon_{\mathrm{smooth}}^{j}={\sum_{k=1}^{D_{2}}\lambda_{j}^{\left(k\right)}\left\|b_{j}^{\left(k\right)}-B_{t}B_{t}^{T}b_{j}^{\left(k\right)}\right\|}^{2}$$

where $\varepsilon_{smooth}^{j}$ denotes the smooth error between the new projection matrix $B_{t}$ and the previous projection matrix $B_{j}$. The weight of each base vector $b_{j}^{\left(k\right)}$ in $B_{j}$ is $\lambda_{j}^{\left(k\right)}\geq 0$; the projection matrix is calculated from the previous frame (*j* < *p*). Using the data term (7) and the smooth term (8), the loss function (6) is minimized under the constraints $B_{t}^{T}B_{t}=I$ to calculate the response score $\hat{y}$ as follows:

(9) $$\hat{y}=F^{-1}(A⊗F(k(z_{i,j},\hat{x}))$$

### 3.2. Color Histogram Feature

The Color Histogram Feature reflects the distribution of the color value of each pixel, which is a type of statistic regarding color information [25,26,27,28]. It describes the proportion of different colors in the whole picture, that is, which colors appear in the statistical image, and the probability of occurrence of various colors. Swain and Ballard first proposed the use of color histograms as representations of image color features. They also pointed out that the color histogram is insensitive to the geometric transformation of the image with the rotation of the axis of view, as well as with the translation and scaling of the amplitude, and color histograms are insensitive to changes in image quality, such as blurring. This property of color histograms makes them more suitable for retrieving the global color similarity of the image, that is, by comparing the differences in a color histogram to measure the difference in the global distribution of the two images. In 2015, another kind of color histogram, DAT, was proposed by Possegger et al. [16]. DAT is a global statistical feature that identifies the potential interference areas in advance, effectively distinguishes between targets and backgrounds, and handles deformation and illumination changes. Therefore, the color histogram feature proposed in this paper adopts the color histogram feature in Ref. [16].

### 3.3. Dual Color Feature Fusion Strategy

CN is a language color label that describes color in human terms, and that describes the color attributes at the pixel level. CN features describe the main color component of the target, which has the characteristics of being insensitive to image size and direction. The CH is also a color feature; however, when compared with the CN feature, it is obviously different. CH is a statistic on the color information of the whole picture, regardless of the specific position of the color in the image, where colors appear in the statistical image and the probability of occurrence of various colors. Regardless of whether the image is scaled, rotated, panned, etc., the color histogram is not affected.

The idea of the dual color feature fusion with dimension-reduced object tracking algorithm (CDPS) proposed in this paper is derived from the 2016 Staple [21] tracker, which combines two color features of CN features and CH features according to the method in the Staple. However, a huge challenge concerns the determination of the proportion of each feature response. The traditional tracking algorithm requires a large number of tuning parameters, through continuous tuning parameters, to find the optimal feature response coefficient. In order to solve the redundant parameter adjustment work, a method of adaptively adjusting the feature weight coefficient of the target scene is proposed.

**Figure 2:**
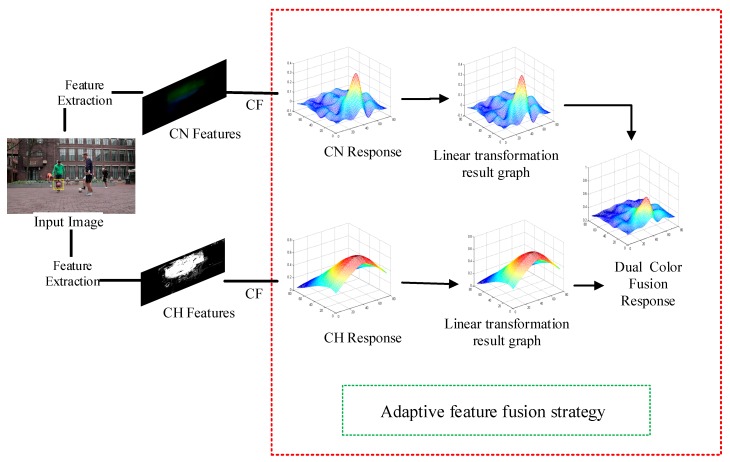
Feature fusion flow chart. CF denotes the Correlation Filter operation.

The feature fusion flow chart of this paper is shown in Figure 2. In that figure, the input image is firstly subject to feature extraction, CN features and CH features are respectively extracted, and then CF processing is performed, i.e., the template and candidate region are convoluted by the CF method to obtain the respective corresponding responses. Then, the adaptive feature fusion strategy is used to adaptively fuse the CN response and the CH response to obtain a dual color fusion response.

According to the method in Ref. [21], a score function of the double-color features $f_{\mathrm{color}}(x)$ is obtained by a linear combination of the CN score $f_{\mathrm{cn}}$ with the color CH score $f_{\mathrm{hist}}$:

(10) $$f_{\mathrm{color}}(x)=\gamma_{\mathrm{cn}}f_{\mathrm{cn}}(x)+\gamma_{\mathrm{hist}}f_{\mathrm{hist}}(x)$$

where, *γ*_cn_ is the weight coefficient corresponding to the CN response; *γ*_hist_ is the weight coefficient corresponding to the CH response.

(1) The score function of the CH is recorded as:
  (11) $$f_{\mathrm{hist}}(x;\beta )=g(\varphi_{x};\beta )$$

This term is $\varphi_{x}:H\to {ℜ}^{M}$, calculated from an M-channel feature image, obtained from image *x*, and defined on the (different) finite grid $H\subset Z^{2}$, where β is the histogram weight vector.

The CH score is invariant for the spatial arrangement of its feature images, so that for any permutation matrix Π,g(φ)=g(Πφ). This paper uses a linear function of a (vector value) average feature pixel, as shown in the following equation:

(12) $$g(\varphi ;\beta )=\beta^{T}(\frac{1}{H}\sum_{u\in H}\varphi \left[u\right])$$

The average form of converting Equation (10) into a scalar fraction image is as follows:

(13) $$g(\varphi ;\beta )=\frac{1}{H}\sum_{u\in H}\zeta_{\left(\beta ,u\right)}\left[u\right]$$

where $\xi_{\left(\beta ,\varphi \right)}[u]=\beta^{T}[u],\varphi [u]$ is a feature pixel. Since the feature transformation and translation have a commutative law $\varphi_{T(x)}=T(\varphi_{x})$, a single integral image can be used to obtain a histogram score, which speeds up the calculation of the convolution operation.

(2) The score function is the response score function, so that the CN score function is:
  (14) $$f_{\mathrm{cn}}(x)=\hat{y}=F^{-1}(A⊗F(k(z_{i,j},\hat{x}))$$
(3) The key step of the feature fusion strategy is how to adaptively obtain the weight coefficients *γ*_cn_ and *γ*_hist_. In the course of the experiment, we found that the color histogram feature weight score was relatively large in any scene. Directly performing the dual color feature fusion to obtain the tracking method is very sensitive to the color attribute, which easily leads to a failure in target tracking. In order to solve this problem, we introduce a suppression term, *μ* to the response of the histogram feature to obtain the final color histogram weight coefficient.

The process of acquiring the adaptive weight coefficient is as follows. Let us $\sigma_{\mathrm{cn}}=\max(f_{\mathrm{cn}}(x))$ make and $\sigma_{\mathrm{hist}}=\max(f_{\mathrm{hist}}\left(x\right))$, then *γ*_cn_ and *γ*_hist_ can be expressed as:

(15a) $$\gamma_{\mathrm{cn}}=\frac{1-\sigma_{\mathrm{cn}}}{\sqrt{\sigma_{\mathrm{cn}}{}^{2}+\sigma_{\mathrm{hist}}{}^{2}}}$$

(15b) $$\gamma_{\mathrm{hist}}=\mu \frac{1-\sigma_{\mathrm{hist}}}{\sqrt{\sigma_{\mathrm{cn}}{}^{2}+\sigma_{\mathrm{hist}}{}^{2}}}$$

Here, $\mu =\frac{\sigma_{\mathrm{cn}}}{\sigma_{\mathrm{hist}}}$ after obtaining the adaptive weight coefficient, and we can determine the final dual color feature response score by Equation (8) and find the maximum response score of the double-color feature to determine the target position of the next frame.

### 3.4. Scale Reduction

#### 3.4.1. Principal Component Analysis 

PCA is a kind of multivariate statistical analysis method based on multidimensional orthogonal linear transformation which is often used to reduce the dimensionality of data and feature extraction of signals [29,30,31]. Its essence is to analyze the main influencing factors from multivariate terms. To reveal the essence of things and simplify complex problems, the projection method that best represents the original data in the sense of the least mean square is found. This projection process is the process of dimension reduction.

PCA is a statistical analysis method that is based on the principle of K-L transformation. After K-L transformation, the sample space can be described by a small number of features. According to the sample matrix X, the covariance matrix $Q=XX^{T}$ is calculated; then, the Q matrix eigenvalues and eigenvectors are calculated, and the eigenvectors corresponding to the larger n eigenvalues are taken to form the feature subspace $W^{T}$. According to $Y=W^{T}X$ the sample X, the description can be reduced from the original R-dimensional space to M-dimensional space (R ≫ M). After the dimension reduction, the main information of the sample is retained, and the data amount is well obtained. The specific model of compression is as follows: 

Given a data set sample point set $X=\{x_{1},x_{2},\ldots ,x_{n}\}$, in that data set, there are *n* sample points and each sample point contain *p* indicators, i.e., $x_{i}\in R^{p},i=1,2,\ldots ,n$, then:

(16) $$X=[\begin{array}{cccc} x_{1} & x_{2} & \ldots & x_{p} \end{array}]=|\begin{array}{cc} x_{11} & x_{12}\\ x_{21} & x_{22}\\ \vdots & \vdots \\ x_{n1} & x_{n2} \end{array}\begin{array}{cc} \ldots & x_{1p}\\ \ldots & x_{2p}\\ \vdots & \vdots \\ \ldots & x_{np} \end{array}|$$

Principal component analysis is a linear combination of the original P indicators to obtain the new *p* comprehensive indicators, namely:

(17) $$y_{i}=w_{1i}x_{1}+w_{2i}x_{2}+\ldots +w_{pi}x_{p},i=1,2,\ldots ,p$$

where $x_{i}$ and $y_{i}$ are N-dimensional. The coefficient $w_{ij}$ needs to satisfy the following three conditions so that the random variable indicators obtained after the transformation are irrelevant with each other, and the variances are successively decreased:

- $y_{i},y_{j}$ are not related, where (i≠j,i,j=1,2,⋯,p);
- The variance of the variable $y_{1}$ is not less than the variance of $y_{2}$, and the variance of the variable is gradually decreasing;
- From the above, a projection matrix of p×p can be obtained:
  $$w_{k1}^{2}+w_{k2}^{2}+\ldots +w_{kp}^{2}=1,k=1,2,\ldots ,p$$
  (18) $$W=|\begin{array}{cc} w_{11} & w_{12}\\ w_{21} & w_{22}\\ \vdots & \vdots \\ w_{p1} & w_{p2} \end{array}\begin{array}{cc} \ldots & w_{1p}\\ \ldots & w_{2p}\\ \vdots & \vdots \\ \ldots & w_{pp} \end{array}|$$

Thus, we obtain:

(19) $$Y=[y_{1},y_{2},\ldots ,y_{p}]=W^{T}X$$

#### 3.4.2. Scale Reduction Strategy

In this paper, the idea of the dimensional dimensionality reduction strategy is mainly derived from fDSST [28]. The computational cost of DSST is dominated by the FFT. In the training and detection steps, each feature dimension requires an FFT. In order to reduce the required number of FFT computations, the dimension reduction strategy of PCA is used to reduce the number of dimensions to 17.

Establishing a scale space CF, we obtain *P_t_* by minimizing the reconstruction error of the target template *μ_t_*, as shown in Equation (18). The projection matrix *P_t_* is *d*_1_ × *d*, where *d*_1_ is the dimensionality of the compressed feature representation:

(20) $$\varepsilon =\sum_{n}||u_{t}(n)-P_{t}^{T}P_{t}u_{t}(n)||$$

Equation (20) is minimized under the orthonormality constraint $P_{t}^{T}P_{t}=I$. A solution is obtained by performing an eigenvalue decomposition of the autocorrelation matrix:

(21) $$C_{t}={\sum_{n}u_{t}(n)u_{t}(n)}^{T}$$

Using the same algorithm of the position filter Equation (1), the response output of the scale space CF is obtained:

(22) $$Y=F^{-1}(A⊗F(\tilde{z},\tilde{x}))$$

PCA dimension reduction technology, which can reduce high dimensions to low dimensions, reduces redundant computation and improves the operation speed and accuracy. Therefore, PCA dimension reduction technology was integrated into the Staple framework. The results of the experiment (shown in Section 4) prove that scale reduction based on dual color fusion is better than direct dual color fusion.

## 4. Experiment

### 4.1. Implementation Details

The experimental platform of this paper is shown in Table 1. In this paper, in addition to the fusion of the characteristic response coefficient in the linear response phase of the characteristic response, other parameter settings retained the same parameters as the original document in order to better verify the effectiveness of the proposed method and avoid the innovation of the method proposed in this paper due to the adjustment problem.

### 4.2. Qualitative Analysis

**Figure 3:**
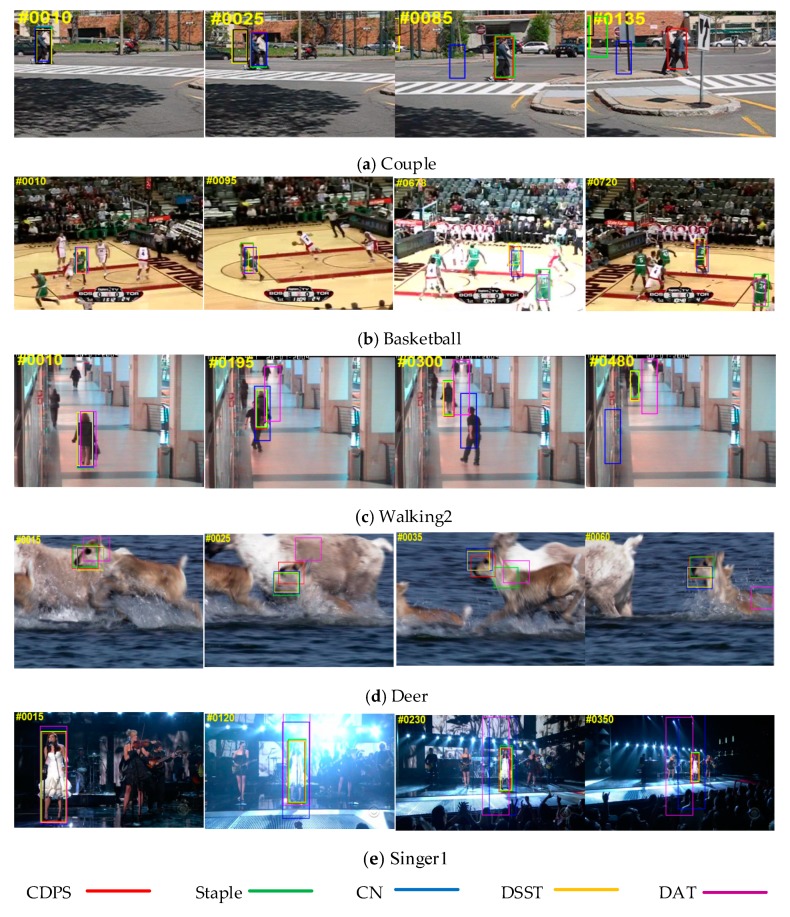
A comparison of our approach with the state-of-the-art trackers Staple, CN, DSST, and DAT. The example frames are from the “Couple”, “Basketball”, “Walking2”, “Deer”, and “Singer1” sequences, respectively. The results of Staple [21], CN [15], DSST [9], DAT [16], and our approach are represented by green, blue, yellow, pink, and red boxes, respectively.

In order to better verify the effectiveness of the proposed algorithm, the algorithm run on the OTB-13 [32], and selected five challenging data sets for verification. These were used to compare with current popular algorithms (Staple [21], DAT [16], CN [15], DSST [9]), as shown in Figure 3. The selected image sequence set attributes are shown in Table 2.

As shown in Figure 3, the algorithm was qualitatively compared with four CF-based tracking algorithms (Staple, DAT, CN, DSST) in the five challenging data sets shown in Table 2. The tracking results are analyzed as follows:

(1) Deformation: Figure 3a. The “Couple” sequence had a deformation in the process of motion. From this sequence, CN and DSST were the earliest tracking failures, and then DAT and Staple deviated from the target position. Tracking the target showed that the tracking method proposed in this paper has the best tracking performance in the target deformation process, and Staple is the second-best tracker.
(2) Occlusion: Figure 3c. The target of the 195th frame in the “Walking2” sequence was obviously occluded, and the DAT had a significant offset. At the 300th frame, the CN also drifted. The proposed algorithm had better robustness.
(3) Fast motion, motion blur: A fast-moving situation is shown in Figure 3d, “Deer” sequence. The target moved quickly during the tracking process. At the 15th frame, the DAT had deviated from the target position at the 25th and 35th frames. The target had moved and blurred. In the figure, it was seen that Staple, CN, DSST, and DAT had large offsets. Only the algorithm did not drift, which indicated that the algorithm had the best tracking performance under fast-moving and moving-blur situations.
(4) Illumination: Illumination changes were shown in the “Basketball” sequence (Figure 3b), and the “Singer1” sequence (Figure 3e). During the tracking process, obvious illumination changes occurred, and DAT found obvious drift. The results prove that the performance of the dual color feature is greatly improved compared to the single color feature tracker in the illumination change scenario.
(5) In-plane rotation and out-of-plane rotation: “Couple” sequence (Figure 3a) and “Basketball” sequence (Figure 3b) produced internal and external rotation changes during the motion. From the “Couple” sequence, it was seen that at the 30th frame the DSST tracker could not keep up. The target, DAT, could not keep up with the 80th frame. At the 140th frame, only the algorithm was left. From the “Basketball” sequence, it was seen that Staple and DAT were much cheaper, and CN and DSST were also less expensive. The comparison results show that the proposed algorithm performs better for internal and external rotation scenes.

### 4.3. Quantitative Analysis

In order to evaluate the performance of the target-tracking algorithm, two important evaluation indicators—Distance Precision (DP) and Overlap Precision (OP)—were used Ref. [32]. The accuracy DP was evaluated by the central position error, which was the Euclidean distance between the center point of the real target frame and the center point of the target frame that was tracked and positioned. The success rate OP was evaluated by the overlap accuracy. The overlap precision refers to the ratio of the intersection of the tracked target frame area and the real target frame area to the union. In the tracking process, if the center error value and the boundary overlap rate satisfied a certain threshold (the DP threshold was usually set to 20, the OP set to 0.5) the tracking was successful.

#### 4.3.1. Quantitative Analysis of Feature Comparison Experiments

**Figure 4:**
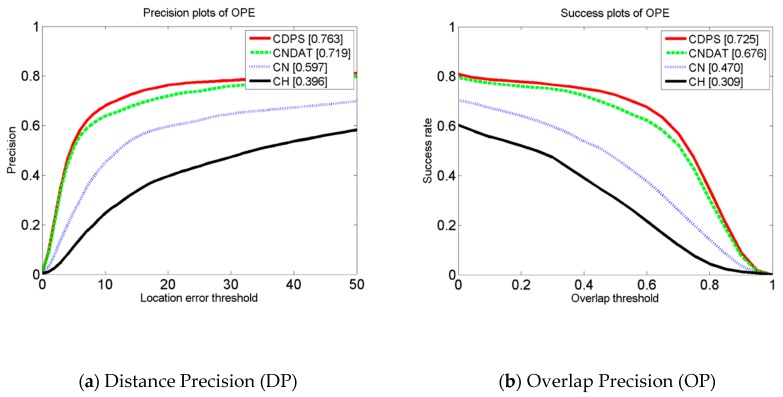
Feature comparison chart.

In this paper, we refer to the proposed method that performed dual color fusion without PCA as CNDAT. In CNDAT, the parameter *γ*_cn_ is set to 0.8 and the *γ*_hist_ is set to 0.2. Meanwhile, the proposed algorithm based on the dual color fusion scale reduction was named CDPS. In the experiment, we compared the simple color feature CN, the color histogram CH, the two-color fusion feature CNDAT, and the final algorithm CDPS, on OTB-13 datasets. The comparison chart is shown in Figure 4. It can be seen from this figure that CDPS performed the best, whether DP or OP were used. For DP (Figure 4a), CDPS was 4.4% higher than CNDAT, 16.6% higher than CN, and 36.7% higher than CH; for OP (Figure 4b), CDPS increased by 4.9% compared with CNDAT, and was 25.5% higher than CN and 41.6% higher than CH. Experiments show that our algorithm was greatly improved in both accuracy and success rate. There were two main reasons for this: first, the feature algorithm that combines the two colors makes up for the lack of a single color feature; second, adding the dimension reduction strategy to reduce the complexity of the algorithm can further improve the performance of the algorithm.

#### 4.3.2. Comparative Analysis of Each Tracking Algorithm

In order to demonstrate the performance of our method, we plotted the experimental results of different challenge attribute sequences on OTB-13, as shown in Table 3 and Table 4. Table 3 and Table 4 respectively show the accuracy and success rate of the four trackers Staple, DAT, CN, and DSST in the qualitative analysis in Section 4.2, and the five data sets of the algorithm in Couple, Basketball, Walking, Deer, and Singer1.

The accuracy rate is obtained from the error between the true annotation value and the center position of the measured value. It can be seen from Table 3 that the tracking method proposed in this paper had the highest accuracy, especially in the cases of occlusion, illumination variation, target external rotation variation, etc. The accuracy is high in the five datasets, which was obviously superior to the comparison algorithm. It was seen that the algorithm was more stable in the above environments.

The overlap rate is obtained from the truth box and the measurement frame through Table 4. In the five data sets tested, the average accuracy of the tracking method proposed in this paper could reach 0.7093, while the average overlap rates of Staple, DAT, CN, and DSST were 0.6963, 0.3428, 0.4448 and 0.6108, respectively. It can thus be seen that the proposed method has the best performance compared with the other four tracking methods. Compared with the single color feature CN and CH performances, the robustness and effectiveness of the proposed algorithm are proven.

#### 4.3.3. Quantitative Analysis of the Dimensional Reduction of PCA Scale

In order to verify performance of the PCA Scale Dimension Reduction, in this experiment, five datasets which are the most representative challenging attributes of scale variation (Trellis, Doll, Dog1, Lemming, Liquor) were selected from the OTB-13 dataset. The real-time ability of the proposed algorithm CDPS was compared with CNDAT (perform Dual Color Feature Fusion Strategy without PCA scale reduction), and the state-of-the-art methods including Staple [21], DAT [18], CN [15], DSST [9]. In Table 5, the average speeds (frame per second, fps) of algorithms running at datasets are presented. It can be seen from Table 5, the CDPS is faster than the CNDAT, indicating that scale reduction reduce the computational cost and improved the running speed; Compared with Staple, DAT, and CN, running speed of the CDPS was lower, but it can still perform a real-time tracking. Meanwhile, in terms of success rates and precision rates, the CDPS outperforms other tracker as shown in Table 3 and Table 4.

#### 4.3.4. Overall Tracking Performance

**Figure 5:**
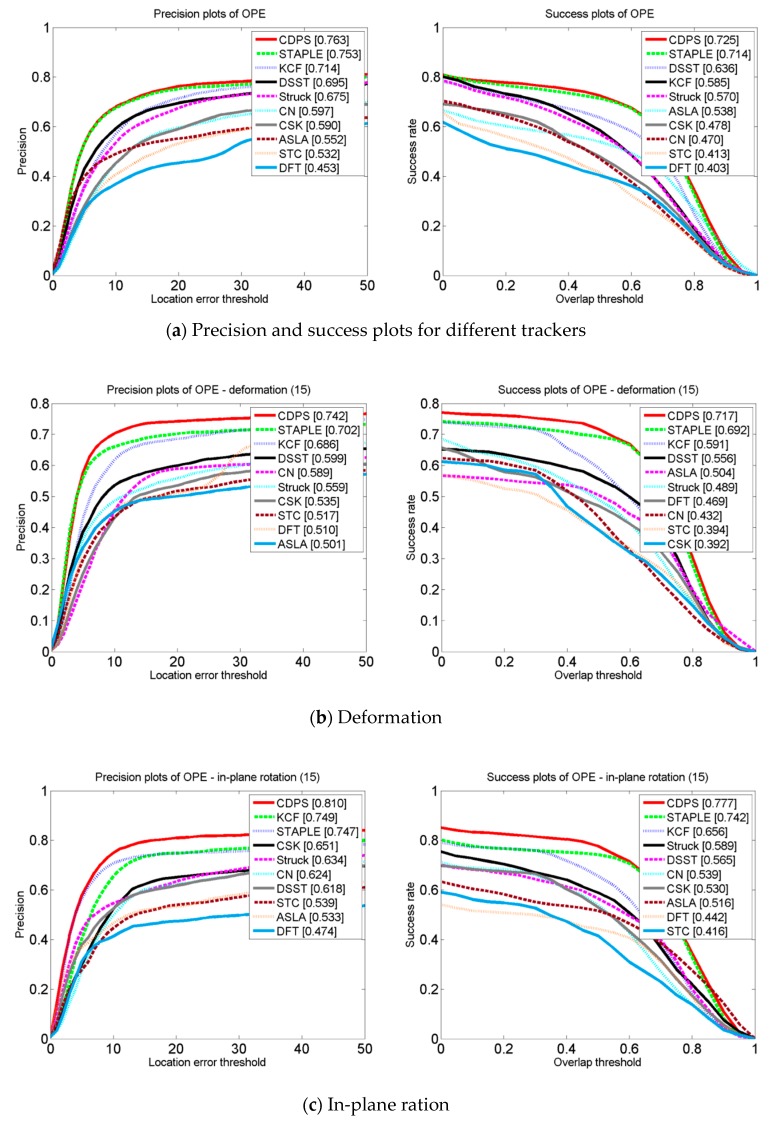

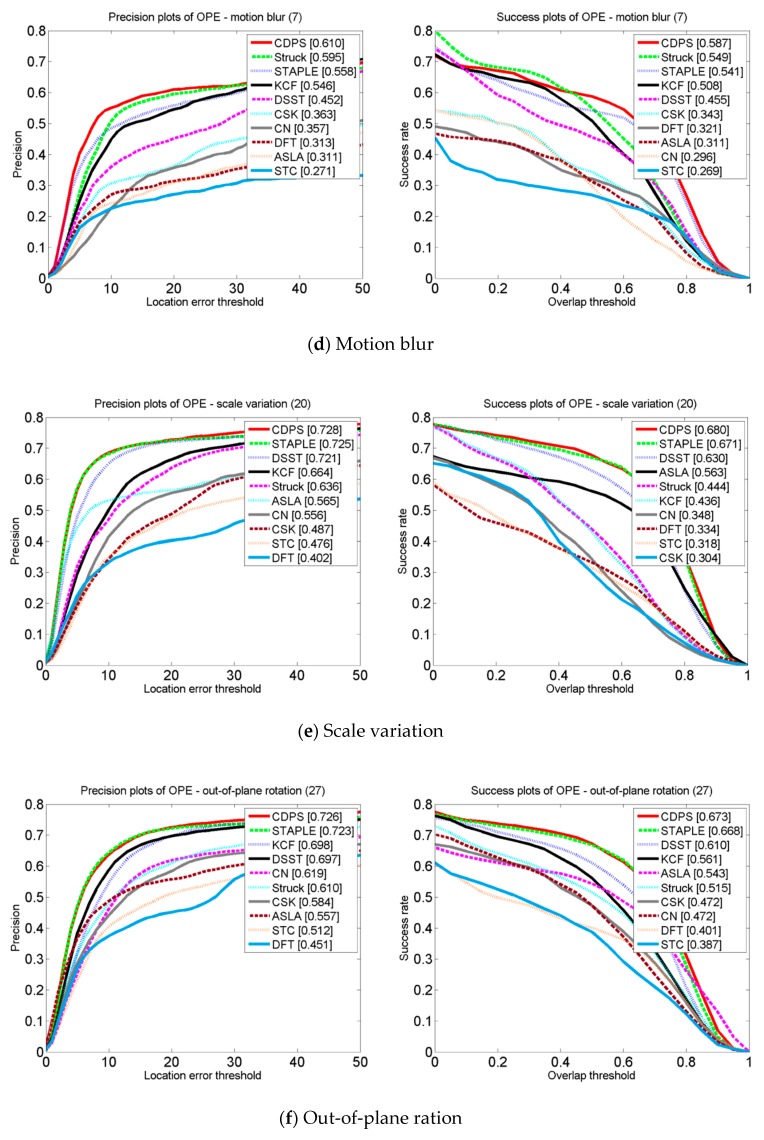

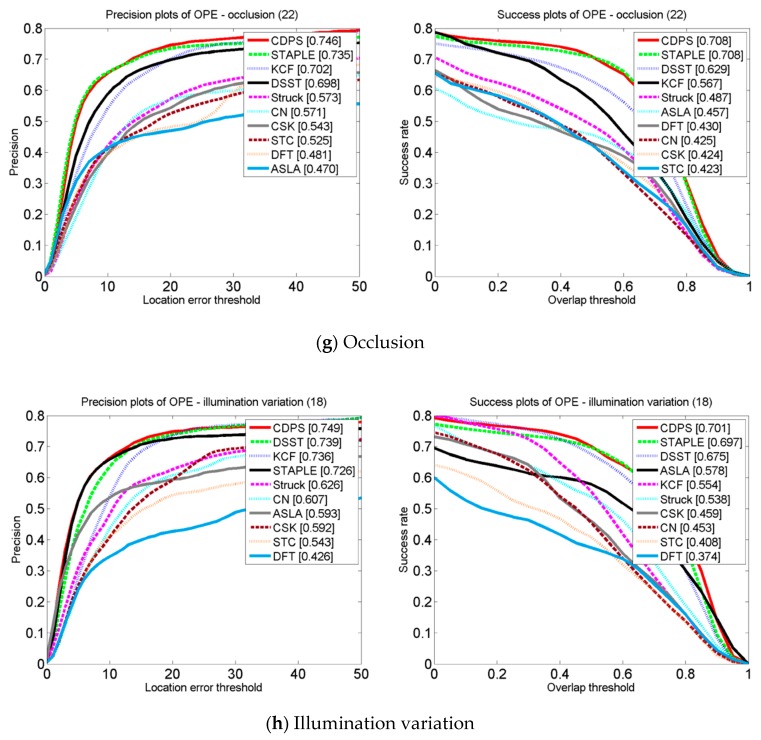
Attribute-based evaluation of precision and success plots comparing algorithms with 10 CF-based trackers over seven challenges of deformation: in-plane ration, scale variation, out-of-plane ration, occlusion, motion blur, and illumination variation. The number of sequences for each attribute is shown in brackets.

Due to the complex scene, the effect of the object tracking algorithm was usually greatly related to the datasets. In order to further prove the effectiveness of the proposed algorithm, extensive experiments were performed on OTB-13 datasets; our method obtained the top rank in performance, outperforming nine state-of-the-art trackers on OTB. Figure 5 shows a graph of the accuracy and success rate of the algorithm and of the nine state-of-the-art trackers on different attribute sequences of the OTB-13. It can be seen from Figure 5 that the algorithm achieved good tracking results on these seven attributes, especially in the target deformation sequence, that the CDPS tracker in this paper was 4% more accurate than Staple, and that the success rate increased by 2.5%. In the motion blur sequence, the CDPS was 5.2% more accurate than Staple, and the success rate improved by 4.6%. In the in-plane ration sequence, the tracker CDPS accuracy improved by 6.3% compared to Staple, and the success rate increased by 3.5%. By analyzing the results in Figure 5, it can be seen that compared with other tracking algorithms, the tracking method proposed in this paper reached the level of current mainstream algorithms in terms of tracking performance.

## 5. Conclusions

In this paper, based on the Correlation Filter framework, two different performance color features, CN features and CH features, are merged. At the same time, the proposed feature response fusion stage adopts the proposed adaptive feature fusion strategy considering the target scene. In order to reduce the complexity and speed up the algorithm, PCA dimension reduction is added on the basis of the original double color fusion. The performance of the proposed tracking algorithm is verified by the OTB-13 public test set, and compared with the state-of-the-art tracking algorithms. The experimental results show that the proposed algorithm performs best in both accuracy and robustness for most complex scenarios, especially in the case of deformation and in-plane rotation. Although the algorithm achieves good tracking results, due to the diversity and complexity of the target-tracking scene, further research is still needed on the depth and breadth. Future research work can start from the following two limitations: (1) The features of CN and CH are typical traditional hand-designed features. One of the main shortcomings of these hand-designed features is that they cannot effectively capture the semantic information of the target, and it is difficult for them to deal with complex scenes. These features have certain limitations in terms of discriminability. While the depth feature is not good enough in real time, it can extract better features. Therefore, the problem of how to effectively combine traditional features with deep features is worthy of further research; (2) In target tracking, when the target is completely occluded for a long time, the robustness of the target is still not good enough. Therefore, the problem of how to solve the long-term occlusion of targets also needs to be further researched.

## Figures and Tables

**Table 1:** Experimental environment.

System	CPU	Frequency	System Type	RAM	Experimental Software
Windows 10	Intel i7-7700K	4.20 GHz	64	16.0 GB	Matlab R2014a

**Table 2:** Experimental videos.

Video	Number of Frames	Main Challenges
Couple	1140	SV, DEF, OPR, IPR
Basketball	725	IV, DEF, OPR, IPR
Walking2	500	OCC, SV
Deer	71	FM, MB
Singer1	351	IV, SV

IV—Illumination Variation; SV—Scale Variation; OCC—Occlusion; FM—Fast Motion; OPR—Out-of-Plane Rotation; IPR—In-Plane Rotation; DEF—Deformation; MB—Motion Blur.

**Table 3:** Precision rates of the different methods.

Characteristic	Staple [21]	DAT [18]	CN [15]	DSST [9]	CDPS
Illumination variation	0.726	0.357	0.607	0.739	0.763
In-plane rotation	0.747	0.427	0.624	0.618	0.810
Scale variation	0.725	0.403	0.556	0.721	0.728
Occlusion	0.735	0.387	0.571	0.698	0.746
Deformation	0.704	0.589	0.589	0.599	0.742
Out-of-plane rotation	0.723	0.395	0.619	0.697	0.726
Distance Precision	0.753	0.396	0.597	0.695	0.763

**Table 4:** Success rates of the different methods.

Characteristic	Staple [21]	DAT [18]	CN [15]	DSST [9]	CDPS
Illumination variation	0.697	0.300	0.453	0.675	0.701
In-plane rotation	0.742	0.385	0.539	0.565	0.777
Scale variation	0.671	0.282	0.348	0.630	0.680
Occlusion	0.708	0.348	0.425	0.629	0.708
Deformation	0.692	0.448	0.432	0.556	0.717
Out-of-plane rotation	0.668	0.294	0.472	0.610	0.673
Overlap precision	0.714	0.309	0.470	0.636	0.725

**Table 5:** Average speeds (frame per second) of the different tracking algorithms.

	Staple [21]	DAT [18]	CN [15]	DSST [9]	CNDAT	CDPS
Trellis	24.4318	33.7101	110.1833	21.2816	20.5840	21.4211
Doll	34.6974	31.6614	111.1300	21.5115	26.7379	27.6085
Dog1	44.2728	99.3431	220.2279	35.3933	34.9900	35.7940
Lemming	26.9557	27.2720	64.6727	12.3505	18.4105	24.0765
Liquor	22.4907	28.6155	35.9686	7.4456	20.9842	22.4102
Average	30.56968	44.1204	108.4365	19.5965	24.34132	26.13898

## References

[1] Smeulders A.W., Chu D.M., Cucchiara R., Calderara S., Dehghan A., Shah M. (2013). Visual Tracking: An Experimental Survey. IEEE Trans. Pattern Anal. Mach. Intell..

[2] Trucco E., Plakas K. (2006). Video Tracking: A Concise Survey. IEEE J. Ocean. Eng..

[3] Tsagkatakis G., Savakis A. (2011). Online Distance Metric Learning for Object Tracking. IEEE Trans. Circuits Syst. Video Technol..

[4] Yilmaz A. (2006). Object tracking: A survey. ACM Comput. Surv..

[5] Zhang X., Yu Q., Yu H. (2018). Physics Inspired Methods for Crowd Video Surveillance and Analysis: A Survey. IEEE Access.

[6] Bolme D.S., Beveridge J.R., Draper B.A., Lui Y.M. Visual object tracking using adaptive Correlation Filters. Proceedings of the 2010 IEEE Computer Society Conference on Computer Vision and Pattern Recognition.

[7] Henriques J.F., Rui C., Martins P., Batista J. (2012). Exploiting the Circulant Structure of Tracking-by-Detection with Kernels. Computer Vision—ECCV 2012.

[8] Henriques J.F., Caseiro R., Martins P., Batista J. (2014). High-Speed Tracking with Kernelized Correlation Filters. IEEE Trans. Pattern Anal. Mach. Intell..

[9] Danelljan M., Häger G., Khan F.S., Felsberg M. Accurate Scale Estimation for Robust Visual Tracking. Proceedings of the British Machine Vision Conference.

[10] Khan F.S., van de Weijer J., Vanrell M. (2012). Modulating shape features by color attention for object recognition. Int. J. Comput. Vis..

[11] van de Weijer J., Schmid C. (2006). Coloring local feature extraction. European Conference on Computer Vision 2006 May 7.

[12] Khan F.S., Anwer R.M., van de Weijer J., Bagdanov A., Lopez A., Felsberg M. (2013). Coloring action recognition in still images. Int. J. Comput. Vis..

[13] Swain M.J., Ballard D.H. (1991). Color indexing. Int. J. Comput. Vis..

[14] Cheng Y. (1995). Mean Shift, Mode Seeking, and Clustering. IEEE Trans. Pattern Anal. Mach. Intell..

[15] Danelljan M., Khan F.S., Felsberg M., Van de Weijer J. (2014). Adaptive Color Attributes for Real-Time Visual Tracking. Proceedings of the 2014 IEEE Conference on Computer Vision and Pattern Recognition.

[16] Possegger H., Mauthner T., Bischof H. (2015). In defense of color-based model-free tracking. IEEE Conference on Computer Vision and Pattern Recognition.

[17] Li P., Li X. (2011). Mean shift tracking algorithm based on gradient feature and color feature fusion. Microcomput. Appl..

[18] Dong W., Yu S., Liu S., Zhang Z., Gu W. (2014). Image Retrieval Based on Multi-feature Fusion. Proceedings of the 2014 Fourth International Conference on Instrumentation and Measurement, Computer, Communication and Control.

[19] Huang M., Shu H., Ma Y., Gong Q. (2015). Content-based image retrieval technology using multi-feature fusion. Optik–Int. J. Light Electron Opt..

[20] Morenonoguer F., Andradecetto J., Sanfeliu A. (2003). Fusion of Color and Shape for Object Tracking under Varying Illumination. Lect. Notes Comput. Sci..

[21] Bertinetto L., Valmadre J., Golodetz S., Miksik O., Torr P.H. (2015). Staple: Complementary Learners for Real-Time Tracking. IEEE Conference on Computer Vision and Pattern Recognition.

[22] Danelljan M., Bhat G., Khan F.S., Felsberg M. (2016). ECO: Efficient Convolution Operators for Tracking. IEEE Conference on Computer Vision and Pattern Recognition.

[23] Li F., Tian C., Zuo W., Zhang L., Yang M.H. (2018). Learning Spatial-Temporal Regularized Correlation Filters for Visual Tracking. arXiv.

[24] Danelljan M., Häger G., Khan F.S., Felsberg M. (2016). Discriminative Scale Space Tracking. IEEE Trans. Pattern Anal. Mach. Intell..

[25] Urban J.P., Buessler J.L., Kihl H. Color histogram footprint technique for visual object tracking. Proceedings of the 2005 IEEE Conference on Control Applications (CCA 2005).

[26] Chen T.M., Luo R.C., Hsiao T.H. Visual tracking using adaptive color histogram model. Proceedings of the 25th Annual Conference of the IEEE Industrial Electronics Society (Cat. No.99CH37029).

[27] Leichter I., Lindenbaum M., Rivlin E. (2010). Mean Shift tracking with multiple reference color histograms. Comput. Vis. Image Understand..

[28] Yan Z., Zhan H.B., Wei W., Wang K. (2006). Weighted Color Histogram Based Particle Filter for Visual Target Tracking. Control Decis..

[29] Dong H., Gao J., Liangmei H.U., WenWen D.O.N.G. (2003). Research on the shape feature extraction and recognition based on principal components analysis. J. Hefei Univ. Technol..

[30] Lkopf B., Smola A.J., Ller K.R. (1997). Kernel principal component analysis. Artificial Neural Networks—ICANN’97.

[31] Zhou J., Xing H.E. (2015). Study on the Evaluation on the Core Journals of Management Science Based on Principle Component Analysis. Sci-Tech Inf. Dev. Econ..

[32] Wu Y., Lim J., Yang M.H. (2013). Online Object Tracking: A Benchmark. Proceedings of the 2013 IEEE Conference on Computer Vision and Pattern Recognition.

